# Combining the fractional order derivative and machine learning for leaf water content estimation of spring wheat using hyper-spectral indices

**DOI:** 10.1186/s13007-024-01224-0

**Published:** 2024-06-22

**Authors:** Zinhar Zununjan, Mardan Aghabey Turghan, Mutallip Sattar, Nijat Kasim, Bilal Emin, Abdugheni Abliz

**Affiliations:** 1 School of Resources and Environment, Yili Normal University, Yining, 835000 China; 2 College of Resources and Environmental Sciences, Xinjiang University, Urumqi, 830046 China; 3 State Key Laboratory of Oasis and Desert Ecology, Xinjiang Institute of Ecology and Geography, Chinese Academy of Sciences, Urumqi, 830011 China; 4 College of Information Management, Xinjiang University of Finance and Economics, Urumqi, 830012 China

**Keywords:** Spring wheat, Leaf water content, Multiband Spectral Index, Fractional order derivative, Machine learning

## Abstract

Leaf water content (LWC) is a vital indicator of crop growth and development. While visible and near-infrared (VIS–NIR) spectroscopy makes it possible to estimate crop leaf moisture, spectral preprocessing and multiband spectral indices have important significance in the quantitative analysis of LWC. In this work, the fractional order derivative (FOD) was used for leaf spectral processing, and multiband spectral indices were constructed based on the band-optimization algorithm. Eventually, an integrated index, namely, the multiband spectral index (MBSI) and moisture index (MI), is proposed to estimate the LWC in spring wheat around Fu-Kang City, Xinjiang, China. The MBSIs for LWC were calculated from two types of spectral data: raw reflectance (RR) and the spectrum based on FOD. The LWC was estimated by combining machine learning (K-nearest neighbor, KNN; support vector machine, SVM; and artificial neural network, ANN). The results showed that the fractional derivative pretreatment of spectral data enhances the implied information of the spectrum (the maximum correlation coefficient appeared using a 0.8-order differential) and increases the number of sensitive bands, especially in the near-infrared bands (700–1100 nm). The correlations between LWC and the two-band index (RVI_1156, 1628 nm_), three-band indices (3BI-3_(766, 478, 1042 nm)_, 3BI-4_(1129, 1175, 471 nm)_, 3BI-5_(814, 929, 525 nm)_, 3BI-6_(1156, 1214, 802 nm)_, 3BI-7_(929, 851, 446 nm)_) based on FOD were higher than that of moisture indices and single-band spectrum, with *r* of − 0.71**, 0.74**, 0.73**, − 0.72**, 0.75** and − 0.76** for the correlation. The prediction accuracy of the two-band spectral indices (DVI_(698, 1274 nm)_ DVI_(698, 1274 nm)_ DVI_(698, 1274 nm)_) was higher than that of the moisture spectral index, with *R*^2^ of 0.81 and *R*^2^ of 0.79 for the calibration and validation, respectively. Due to a large amount of spectral indices, the correlation coefficient method was used to select the characteristic spectral index from full three-band indices. Among twenty seven models, the FWBI-3BI_− 0.8 order_ model performed the best predictive ability (with an *R*^2^ of 0.86, RMSE of 2.11%, and RPD of 2.65). These findings confirm that combining spectral index optimization with machine learning is a highly effective method for inverting the leaf water content in spring wheat.

## Introduction

Water is the main medium for transporting mineral nutrients and conducting physiological and biochemical reactions in plants [1–3]. Lack of water can lead to weakened plant transpiration, hindered mineral transport, and decreased chlorophyll content, and ultimately, it restricts the accumulation of assimilates [4, 5]. Spring wheat can be divided into three stages: early stage (emergence and tillering), middle stage (jointing and booting), and late stage (heading and maturing) [6, 7]. During the heading stage, wheat grains begin to develop rapidly, absorbing nutrients and forming components such as starch and protein [8]. It is necessary to enhance irrigation, maintain soil moisture, and apply fertilizer in a timely manner to promote wheat growth [9]. The water content in the internal tissues of leaves can reflect information about soil moisture, crop growth and development, disease resistance, and other factors [10, 11], making it an important indicator for evaluating crop growth and development [12]. Therefore, obtaining real-time and accurate information on leaf water content (LWC) in field crops has important guiding significance for assisting regional agricultural production management.

With the development of remote sensing techniques, remotely sensed data have been widely used to accurately and non-destructively monitor crop parameters [13, 14]. Hyperspectral had the characteristics of multiple bands, strong continuity, and large information; and was performed accurately and quickly with the following objectives: diagnosis of crop leaf water, nutrient status, monitoring of crop growth, spatial variation information of crop biochemical components and evaluation of crop yield [15, 16]. Hyperspectral remote sensing-based modeling can utilize visible and near-infrared reflectance (Vis–NIR) spectra to rapidly respond to changes in crop parameters [17]. However, Hyperspectral data are characterized by a large amount of data and multicollinearity and are usually composed of three types of spectral information: valid information, redundant information, and invalid information [18, 19]; and spectral preprocessing constitutes an important step in spectral modeling analysis. Several spectral preprocessing methods, including spectral continuum removal, spectral logarithmic, first/second-order derivative, and reciprocal logarithmic, and to improve prediction of crop parameters [20]. In general, integer differentials (first/second-order derivative) are mainly used to reduce the influence of baseline drift and isolate overleaping peaks. However, the integer differentials fail to account for some subtle details regarding the reflectance spectra [21, 22]. In contrast, fractional-order derivatives (FOD) not only refine the spectral spacing and amplify weak spectral absorption characteristics in a small interval but also reflect changes in spectral information to some extent [74, 75]. Furthermore, several studies analyzed the relationship between crop components and hyperspectral data based on integer differentials, identified spectral absorption regions (approximately 970 nm, 1200 nm, 1450 nm, 1940 nm, and 2500 nm) of crop leaf moisture, followed by the selection of sensitive bands to predict LWC using partial least square regression (PLSR), Support vector regression (SVR), linear or non-linear functions, and their accuracy is validated [23, 24]. However, there is a lack of accurate research on the simulation models of LWC based on FOD.

The use of spectral indices is a simple and effective method for measuring surface properties, and the band optimization algorithm is widely used in the development of hyperspectral techniques [12, 49]. Compared to single sensitive spectral data, this method has the ability to provide more spectral features and enhance the relationship between crop parameters and spectral features [25]. The spectral index constructed by combining two sensitive bands can improve the accuracy of crop parameter estimation [26, 27]. For example, the ratio of *R*_1600 nm_/*R*_820 nm_ was significantly correlated with the effective moisture thickness of leaves [28, 29], while the application of *R*_1450 nm_/*R*_1940 nm_ can provide a better estimate of plant water status [30]. The depth and area of the absorption valley in the spectral curve are more sensitive to the water content of the crop leaves, and there is a linear positive correlation between the characteristic absorption depth and the area of water near 1450 nm [31, 32]. *DR*_1647 nm_/*DR*_1133 nm_ and *DR*_1653 nm_/*DR*_1687 nm_ are based on derivative spectra and have higher fitting accuracy with crop leaf water compared with that of single band indices [33]. It was demonstrated that combining two bands within the 400–2400 nm range to construct spectral indices (such as RVI, NDVI, and DVI) can improve sensitivity to moisture levels in wheat leaves and enhance the modeling accuracy [47, 49]. However, in practice, the band determination of the sensitivity index depends on the two-dimensional contour map, so the band optimization algorithm is mostly limited to the two-band index form [48]. In previous studies, few researchers have considered extending the spectral index method to more than two bands, particularly optimizing three-band indices and estimating crop leaf moisture by combining traditional spectral indices. Therefore, there is a need to further explore the integrated index based on the fusion of multiband spectral indices.

Remote sensing technology and its integration with machine learning are examples of effective and low-cost acquisition, particularly in the field of environmental studies and earth sciences for optimal management [69]. Several studies have been conducted on hyperspectral remote sensing and inversion models. For instance, Felegari et al., mapped the Cd concentration and introduced the most suitable regression models, including support vector regression (SVR), partial least square regression (PLSR), and artificial neural networks (ANN) [70]. A Wavelet-Attention convolutional neural network (WA-CNN), Random forest (RF) and support vector machine (SVM) algorithms were utilized to automatically map the crops over the agricultural lands [71]. Seyed et al., proposed the two architectures: the first model includes 2D-CNN, skip connections, and LSTM-Attentions and the second model comprises 3D-CNN, skip connections, and Conv-LSTM Attention (The Input data given from MODIS products) [72]. Many strategies have been used to evaluate agricultural products, such as Deep-Yield, CNN-LSTM, and Conv-LSTM. And it showed that machine learning is widely applied in remote sensing technology. However, In the above-mentioned research, there are more studies on the application of machine learning in multi-source remote sensing image, while there are fewer applications of high-precision modeling in ground-based hyperspectral.

The LWC and hyperspectral data were acquired on heading stage of spring wheat. The hypothesis of this study was that band-optimization algorithm using fractional order derivative (FOD) was still effective, and thus, the indices (two-band index and three-band index) calculated based on well processing spectrum will better predict LWC. The combined indices (spectral, two-band index and three-band index) will help to improve the accuracy of predictions of LWC values with the machine learning approaches at heading stage of spring wheat [31, 34].

In this study, twenty seven different models were established separately based on multiband spectral data (single bands, moisture indices, two-band spectral indices and three-band spectral indices) and machine learning (KNN, ANN and SVR). The main purpose of this investigation was to provide a future reference for hyperspectral monitoring of spring wheat leaf moisture under similar production conditions. To achieve this purpose, the major sub-goals were defined:

1. generate and analyze the spectral indices with FOD (with an interval of 0.2, ranging from 0 to 2) for LWC of spring wheat;
2. to evaluate the performance of newly developed spectral indices and combination spectral indices (spectral, moisture indices, two-band index and three-band index) based on FOD for LWC at heading stage of spring wheat;
3. to compare the ability of twenty seven models established by the machine learning approaches to monitor LWC and to identify the optimal model among them.

## Materials and methods

### Location of the study area

**Fig. 1:**
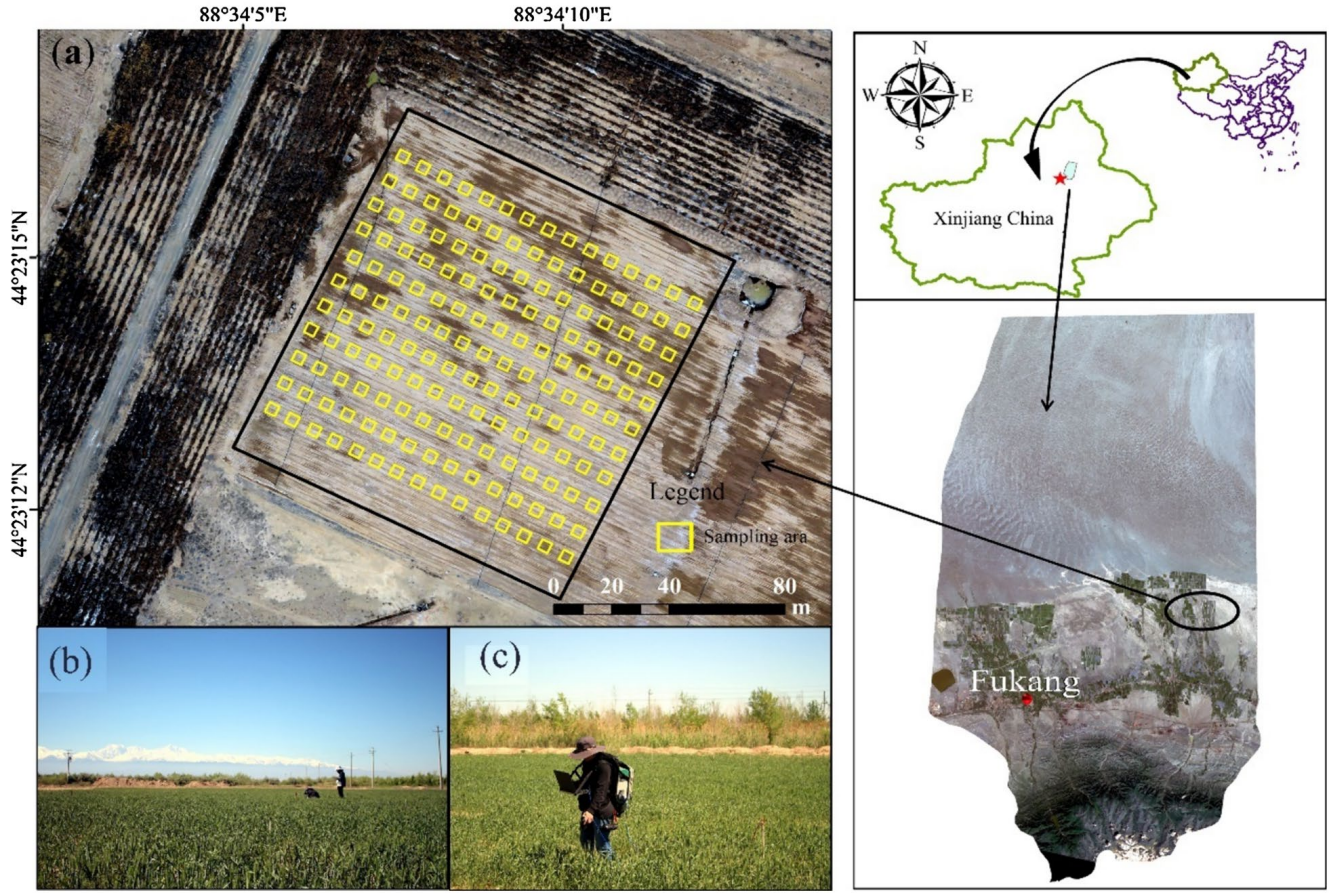
Geographical location of the study area and planting region for spring wheat. **a** Sampling area for field data. **b**, **c** Was spectral data collection

The study was carried out in Fu-kang City, Xinjiang China, situated at an altitude of 577 m and characterized by a temperate continental dry climate, the study sites are depicted in Fig. 1. The experimental site is a typical arid farming area, and the site experiences an average annual temperature of 6.7 °C and an annual precipitation of 205 mm. During the summer season, temperatures are exceptionally high, with significant diurnal variations [34]. In the experimental site, sowing of spring wheat commenced on April 20, 2017 (day of the year (DOY) 96), utilizing 225 kg of seeds per hectare and 17 cm row spacing. Diammonium phosphate (150 kg/ha), ammonium sulfate (150 kg/ha), and potash fertilizer (105 kg/ha) were applied during sowing. At the three-leaf stage, fertilized urea (20 kg) was applied, along with drip irrigation and 750 to 900 m^3^/ha of irrigation in response to rainfall. Chemical methods were employed to remove weeds from the field, 100 ml of 20% Bromoxynil octanoate E.C. was sprayed before jointing stage of the spring wheat (from the 2-leaf stage) [34].

### Field data collection and LWC determination

**Fig. 2:**
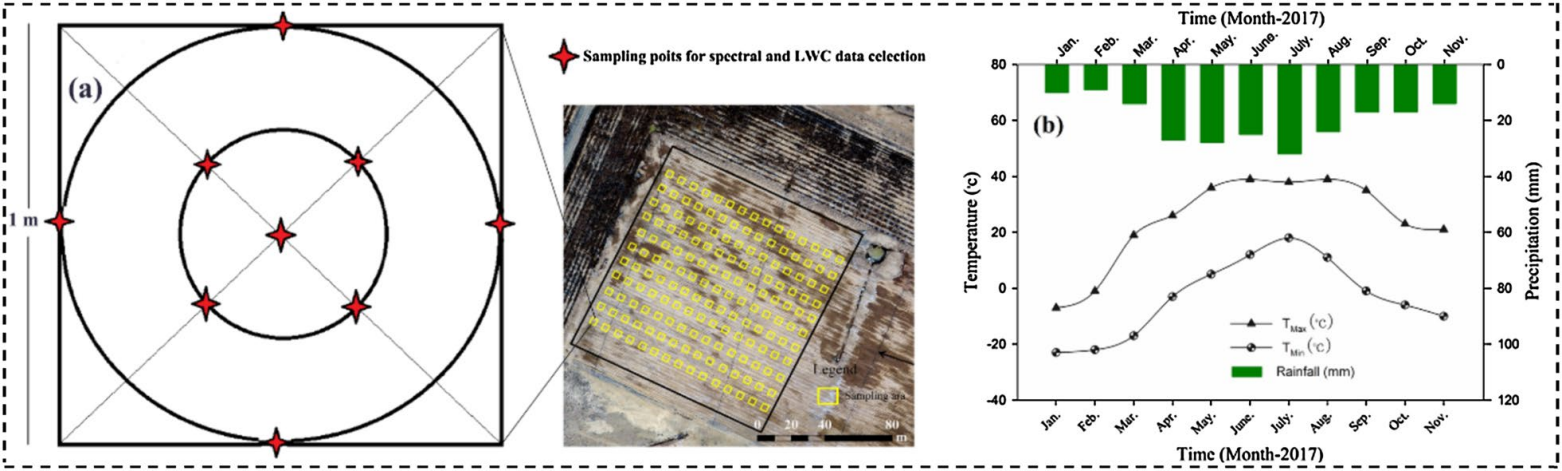
Sample design and climate change. **a** Design of sampling points for field data in the study area. **b** For the 1-year study, daily average precipitation, maximum and minimum air temperature, and precipitation data were collected

To ensure consistency in the measurements, the same treatments were maintained throughout the growing season, and a drip irrigation system was used to determine the timing and amount of water applied. The research area consists of 154 sampling plots, the size of each measuring plot is 1 m × 1 m. Measurements were taken at nine distinct points within each plot (as illustrated in Fig. 2a). Daily average precipitation, maximum and minimum air temperature were collected in the field over the course of a year (as illustrated in Fig. 2b). In 2017, the highest daily average precipitation occurred in July, reaching 38 mm. The highest daily temperature occurred in June and August, while the lowest temperature occurred in January. The lowest temperature in June was 11℃.

We obtained plant samples (on June 04, 2017) from each small sampling area in sealed plastic bags and ensured that plant moisture was not lost. In the laboratory, the leaves and stems of spring wheat were separated, and the weight of each sample was measured with an electronic balance with a sensitivity of 0.0001 g and recorded as the fresh weight [35]. Then, all the samples were dried in an oven (80 ℃) for 36 h and weighed, and the dry weight was recorded. The 154 samples were randomly divided into two groups: one was a modeling sample (70%), which was used to establish a spring wheat leaf moisture content prediction model; the other was a verification sample (30%), which was used to verify the established estimation model [36]. The leaf water content was calculated according to formula (1).

1 $$LWC=\frac{FW-DW}{FW}\times 100\text{\%},$$

where *FW* and *DW* are the fresh and dry weight (g) of spring wheat leaves, respectively.

### Hyperspectral measurement and preprocessing

Spectral reflectance data were collected using the American ASD FieldSpec3 spectrometer, with a band range of 350–2500 nm [37]. The sampling interval was 1.4 nm for the range of 350–1000 nm and 2 nm for the range of 1000–2500 nm [38]. Data collection was carried out in cloudless and sunny weather, the collection time was 10:00 am–02:00 pm, and whiteboard correction was performed every 3–5 min [34]. Ten spectral curves were collected for each sample with a measurement interval of 0.1 s. The average value of these spectral curves was used as the spectral data for that particular sample.

**Fig. 3:**
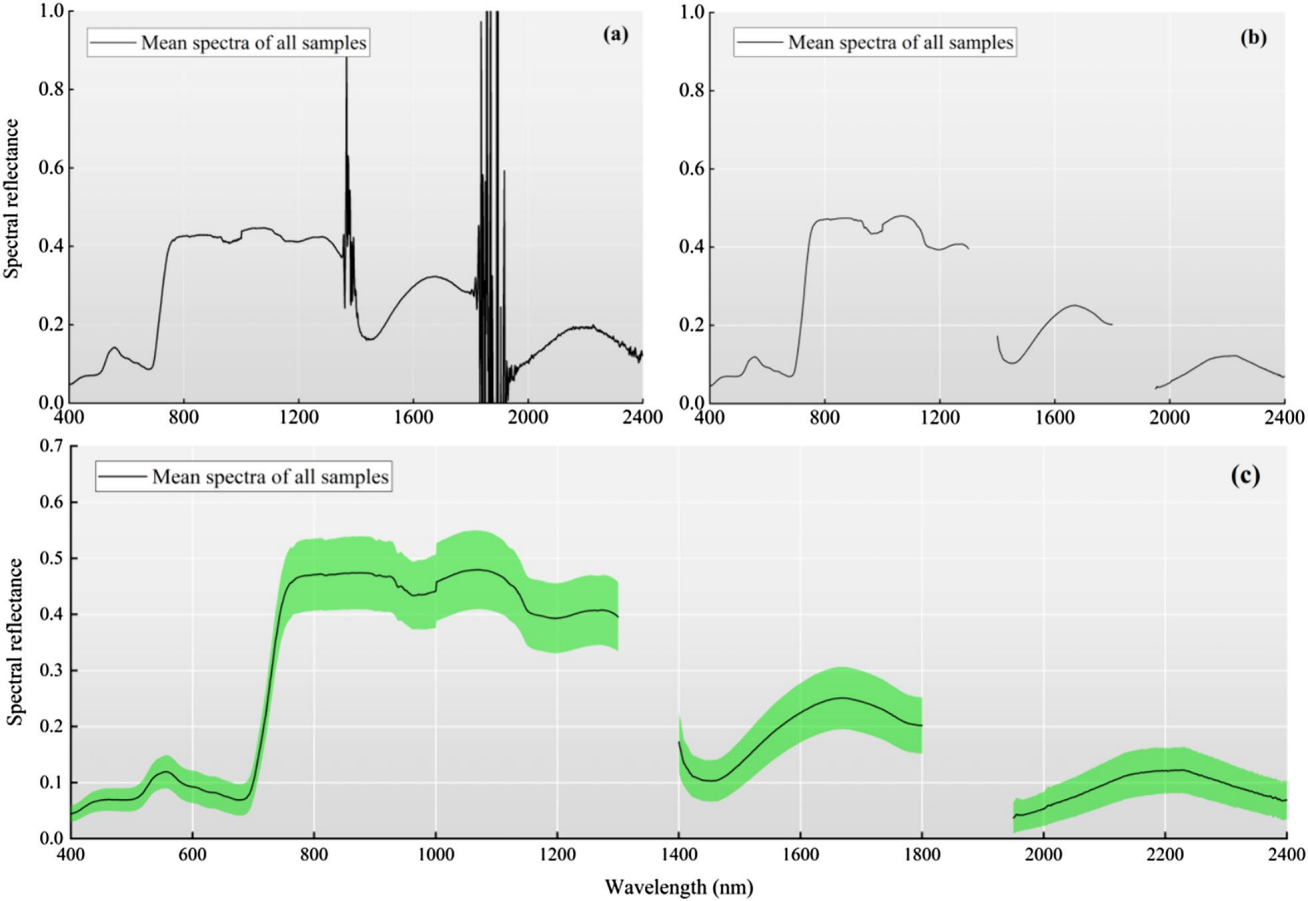
Preprocessing steps for hyperspectral data. **a** Mean raw spectra of all spring wheat leaf samples (n = 154) and (**b**) removal of the external disturbance spectra of all spring wheat samples. **c** The green shaded areas represent the standard deviations of the spectra

The measured spectral data are preprocessed by removing the noisy edge bands (350–399 nm and 2401–2500 nm) as well as the infrared bands (1355–1444 nm and 1777–1949 nm). Then, the remaining spectral curves are smoothed using mathematic morphological filtering [39], as shown in Fig. 3. Finally, the processed spectral data are used as the basis for calculating the spectral indices in the subsequent step.

### Fractional order derivative

The concept of fractional order derivative (FOD) is an extension of the idea of integer order derivatives. This field is dedicated to studying the properties and applications of derivatives with arbitrary orders [40, 41], as noted by Ortigueira et al. in 2011 and Pan et al. in 2013. The FOD method has been successfully employed in system modeling, signal filtering, and pattern recognition. There are three main types of FOD algorithms: Riemann–Liouville (R–L), Grünwald–Letnikov (G–L), and Caputo [42], as described by Saadia et al. in 2016.

The Grünwald–Letnikov (G–L) definition is relatively simple and was used in our study. In general, the first derivative of a function is defined as follows:

2 $$f^{\prime}\left(x\right)=\underset{h\to 0}{\text{lim}}\frac{f\left(x+h\right)-f\left(x\right)}{h}.$$

where *h* is the increment of the independent variable *x*. Then the second derivative of function can be defined as follow:

3 $$f^{\prime\prime}\left(x\right)=\underset{h\to 0}{\text{lim}}\frac{f^{\prime}\left(x+h\right)-f^{\prime}\left(x\right)}{h}=\underset{h\to 0}{\text{lim}}\frac{f\left(x+2h\right)-f2\left(x+h\right)+f\left(x\right)}{{h}^{2}}.$$

If the derivative order of the function $f\left(x\right)$ is increased to the higher order $\left(v\right)$, then the vth derivative order of the function $f\left(x\right)$ can be expressed as:

4 $${f}^{v}\left(x\right)=\underset{h\to 0}{\text{lim}}\frac{1}{{h}^{v}}{\sum_{m=0}^{v}(-1)}^{m}\left(\genfrac{}{}{0pt}{}{v}{m}\right)f\left(x-mh\right).$$

By substituting the Gamma function into the binomial coefficient and extending the fractional order to non-integer orders, we can obtain the G-L formula for the v-order fractional derivative in the interval [a, b].

5 $${f}^{v}\left(x\right)=\underset{h\to 0}{\mathit{lim}}\frac{1}{{h}^{v}}{\sum_{m=0}^{[(b-a/h)]}(-1)}^{m}\frac{\Gamma (v+1)}{m!\Gamma (v-m+1)}f\left(x-mh\right),$$

where *h* is the step length and is set to 1 in this study, and [$(b-a/h)$] is the integer part of $(b-a/h)$*.* Which can be converted to:

6 $$\frac{{d}^{v}f\left(x\right)}{{dx}^{v}}\approx f\left(x\right)+\left(-v\right)f\left(x-1\right)+\frac{\left(-v\right)\left(-v+1\right)}{2}f\left(x-2\right)+\cdots \frac{\Gamma \left(-v+1\right)}{m!\Gamma \left(-v+m+1\right)}f\left(x-m\right).$$

To develop spectra-FOD, Eq. (5) was implemented using MATLAB R2014a (The MathWorks Inc.: Natick, MA, USA). The value of *v* was set to values between 0 and 2 in increments of 0.2 at each step. It is noteworthy that *v* = 0 indicated that the raw reflectance was not processed.

### Spectral index selection

The spectral index comprises several narrow or wide bands combined through some mathematical transformation, which not only considers the interaction between bands but also improves the response to the measured attributes to some extent. Previous studies have mostly used two-band spectral indices for environmental modeling and attribute quantification. Based on previous studies, two-band water vegetation indices sensitive to water content in crop leaves were collected. The 12 spectral indices selected in this study are listed in Table 1.

**Table 1:** Traditional two-band combined spectral index

Moisture index	Abbreviation	Equation	References
Water index	WI	*R*_900 nm_/*R*_970 nm_	[43]
Water band index	WBI	*R*_970 nm_/*R*_900 nm_	[44]
Floating position water band index	FWBI	*R*_900 nm_/Min(*R*_930 nm-980 nm_)	
Simple ratio water index-1	SRW-1	*R*_858 nm_/*R*_1240 nm_	
Simple ratio water index-2	SRW-2	*R*_1070 nm_/*R*_1340 nm_	
Moisture stress index	MSI	*R*_1600 nm_/*R*_820 nm_	[45]
Moisture stress index-1	MSI-1	*R*_870 nm_/*R*_1350 nm_	[44]
Normalized different infrared index	NDI-1	(*R*_850 nm_ − *R*_1650 nm_)/(*R*_850 nm_ + *R*_1650 nm_)	
Normalized different vegetation index	NDVI	(*R*_858 nm_ − *R*_648 nm_)/(*R*_858 nm_ + *R*_648 nm_)	
Normalized different water index-1	NDWI	(*R*_858 nm_ − *R*_2130 nm_)/(*R*_858 nm_ + *R*_2130 nm_)	
Normalized different water index-1	NDWI-Hyp	(*R*_1070 nm_ − *R*_1200 nm_)/(*R*_1070 nm_ + *R*_1200 nm_)	[46]
Normalized different mass index	NDMI	(*R*_1649 nm_ − *R*_1722 nm_)/(*R*_1649 nm_ + *R*_1722 nm_)	[47]

R represents spectral reflectance, λ stands for wavelength

The 12 moisture indices listed in Table 1 are commonly used and are sensitive spectral indices for assessing the water content in crop leaves. These spectral indices have fixed bands and serve as the reference for comparing and analyzing the performance of optimized band combinations in this study. The main focus is on assessing the sensitivity strength between traditional spectral indices and band-optimized spectral indices.

Recent studies have calculated the correlation coefficients between two given bands (λ_1_ and λ_2_) in the Vis–NIR range and the attributes to be measured and have displayed the results visually [48, 49]. This two-dimensional correlation analysis method is beneficial for visualizing the external response and internal meaning of spectra. For example, the normalized difference vegetation index (NDVI) places the strongest reflection band and the weakest reflection band in the numerator and denominator, respectively, and further enlarges the gap between the bands by a normalized ratio operation to maximize the sensitivity of the attributes of the objects to be measured [50, 51].

The addition of a third band in a specific sensitive region to the two-band spectral index can often improve the accuracy of the index estimation, enhance anti-interference ability, and eliminate the saturation phenomenon of commonly used two-band indices [52, 53]. In this study, we used the entire dataset and band optimization algorithm to determine the best wavelength combination. In Table 2, R_λ1_, R_λ2_, and R_λ3_ represent the spectral reflectance of bands λ_1_, λ_2_, and λ_3_ in the range of 400–2400 nm.

**Table 2:** The vegetation index of different bands and multiple combinations

Type	Spectral index	Equation	References
Two-band index (2BI)	Ratio vegetation index (RVI)	R_λ1_/R_λ2_	[54, 55]
	Normalized difference vegetation index (NDVI)	(R_λ1 − _R_λ2_)/(R_λ1+_R_λ2_)	
	Difference vegetation index (DVI)	R_λ1_ − R_λ2_	
Three-band index (3BI)	3BI-1	R_λ1_/(R_λ2×_R_λ3_)	[56, 57]
	3BI-2	R_λ1_/(R_λ2+_R_λ3_)	
	3BI-3	(R_λ1 − _R_λ2_)/(R_λ2+_R_λ3_)	
	3BI-4	(R_λ1 − _R_λ2_)/(R_λ2 − _R_λ3_)	
	3BI-5	(R_λ2+_R_λ3_)/R_λ1_	
	3BI-6	(R_λ1 − _R_λ2_)/[(R_λ1 − _R_λ2_) − (R_λ2 − _R_λ3_)]	
	3BI-7	(R_λ1 − _R_λ2_) − (R_λ2 − _R_λ3_)	

R represents spectral reflectance, λ stands for wavelength

In order to calculate spectral indices in two bands and three bands in batch, we have designed an index calculation software based on the Java environment. The software version is 1.0, and the registration number is 2018Sr281300.

### Overall workflow


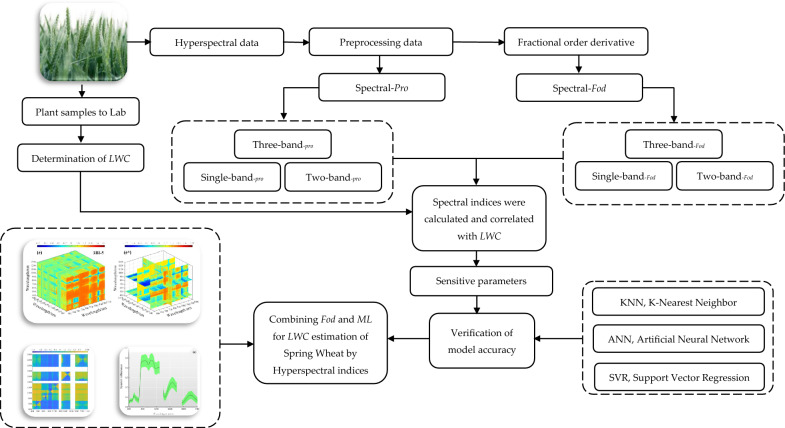


In this study, the FOD was used for leaf spectral processing, and developed new two-band and three-band indices using the band-optimization algorithm, and collectively referred to multiband spectral indices (MBSIs). Additionally, an integrated index is created by combining traditional water indices (MIs) with MBSIs to explore whether the combination of different spectral indices improves the accuracy of estimating LWC.

### Calibration strategies

The K-nearest neighbor KNN algorithm is used in a variety of classification and regression tasks in machine learning. The key idea behind its machine learning applications is that points tend to share the properties of nearby points [10] (the distance function from one point to another often depends on the context-some common ones include Euclidean distance between particles in space, Hamming distance between words, etc.) In a regression setting (where regression is a machine learning technique commonly used to obtain continuous outputs as opposed to discrete outputs in classification), an average (or maximum or minimum) of the KNN is typically used to determine the value of the variable being regressed [76].

Explanation of how KNN works is discussed below: (1) Selecting the optimal value of K; (2) Calculating distance; (3) Finding Nearest Neighbors; (4) Voting for Classification or Taking Average for Regression. In the regression problem, the class label is calculated by taking average of the target values of K nearest neighbors. The calculated average value becomes the predicted output for the target data point.

Artificial neural network (ANN) is a powerful tool used in computer science to solve machine learning problems [58]. It is commonly used for regression and classification tasks. ANN models simulate the electrical activity of the brain and nervous system. In generally, it can fit any non-linear function through a reasonable network structure configuration [59, 77].

ANNs seek to replicate the capabilities of biological neural networks. A node is used to describe an artificial neuron. Like its biologic counterpart, these nodes receive input from synapses and send output when a weight is exceeded [60]. Single-layer ANNs have one layer of input nodes; multilayer ANNs have multiple layers of nodes, including hidden nodes. Both single and multilayer artificial neural networks eventually trigger an output node to fire: this output node makes the decision.

Support vector regression (SVR) is a type of support vector machine (SVM) that is used for regression tasks. It tries to find a function that best predicts the continuous output value for a given input value [61, 62]. The basic steps for building an SVR model are as follows: (1) Data preparation: Collect and preprocess the training data, including feature selection, data cleaning, and normalization; (2) Feature scaling: Scale the input features to ensure they have similar ranges and magnitudes. Common techniques include standardization or normalization; (3) Model selection: Choose an appropriate SVR variant and kernel function; (4) Model training: Use the training data to estimate the model parameters. SVR uses a subset of the training data, called support vectors, to define the regression line or hyperplane; (5) Hyperparameter tuning: Optimize the hyperparameters of the SVR model to improve its performance; (6) Model evaluation: Assess the performance of the trained SVR model using appropriate evaluation metrics.

### Verification of model accuracy

Commonly used evaluation indicators for model estimation capability include coefficient of determination (*R*^2^), root mean square errors (RMSE), and relative percent deviation (RPD).

1. A high coefficient of determination (*R*^2^), indicating a strong linear relationship.
2. Low Root Mean Square Errors (RMSE) of the model’s variables, indicating that the low error between measured and predicted data.
3. Relative Percent Deviation (RPD), indicating the predictive ability of the model. Its computation process is the ratio between standard deviation (SD) and standard error of prediction (SEP). According to the predictive ability of the model, the RPD is divided into three categories: (I) The value of RPD exceeds 2.0, indicating a model with better predictive ability. (II) The RPD values ranging from 1.4 to 2.0 represent a model with general predictive ability. (III) The RPD value is less than 1.4, indicating that it has poor predictive ability.

A model with an *R*^2^ approaching 1, an RMSE approaching 0, and an RPD greater than or equal to 2.0 exhibits estimation ability and stability [49].

$$R^{2}={\left[\frac{\sum_{i=1}^{N}({x}_{i}-\overline{x })({y}_{i}-\overline{y })}{\sqrt{\sum_{i=1}^{N}{({x}_{i}-\overline{x })}^{2}+\sum_{i=1}^{N}{({y}_{i}-\overline{y })}^{2}}}\right]}^{2},$$

$$\text{RMSE}=\sqrt{\frac{\sum_{\text{i}=1}^{\text{N}}{({\upgamma }_{\text{i}}-{\upbeta }_{\text{i}})}^{2}}{\text{n}}},$$

$$RPD\hspace{0.17em}=\hspace{0.17em}SD/SEP.$$

Note: ${x}_{i}$ and ${y}_{i}$ are measured and predicted values, respectively; *x̅* and *y̅* represent the means measured and predicted values, respectively; and n is the number of samples. SD and SEP represent the standard deviation and Standard error of prediction, respectively.

## Results

### Leaf water content and FOD hyperspectral curves

**Fig. 4:**
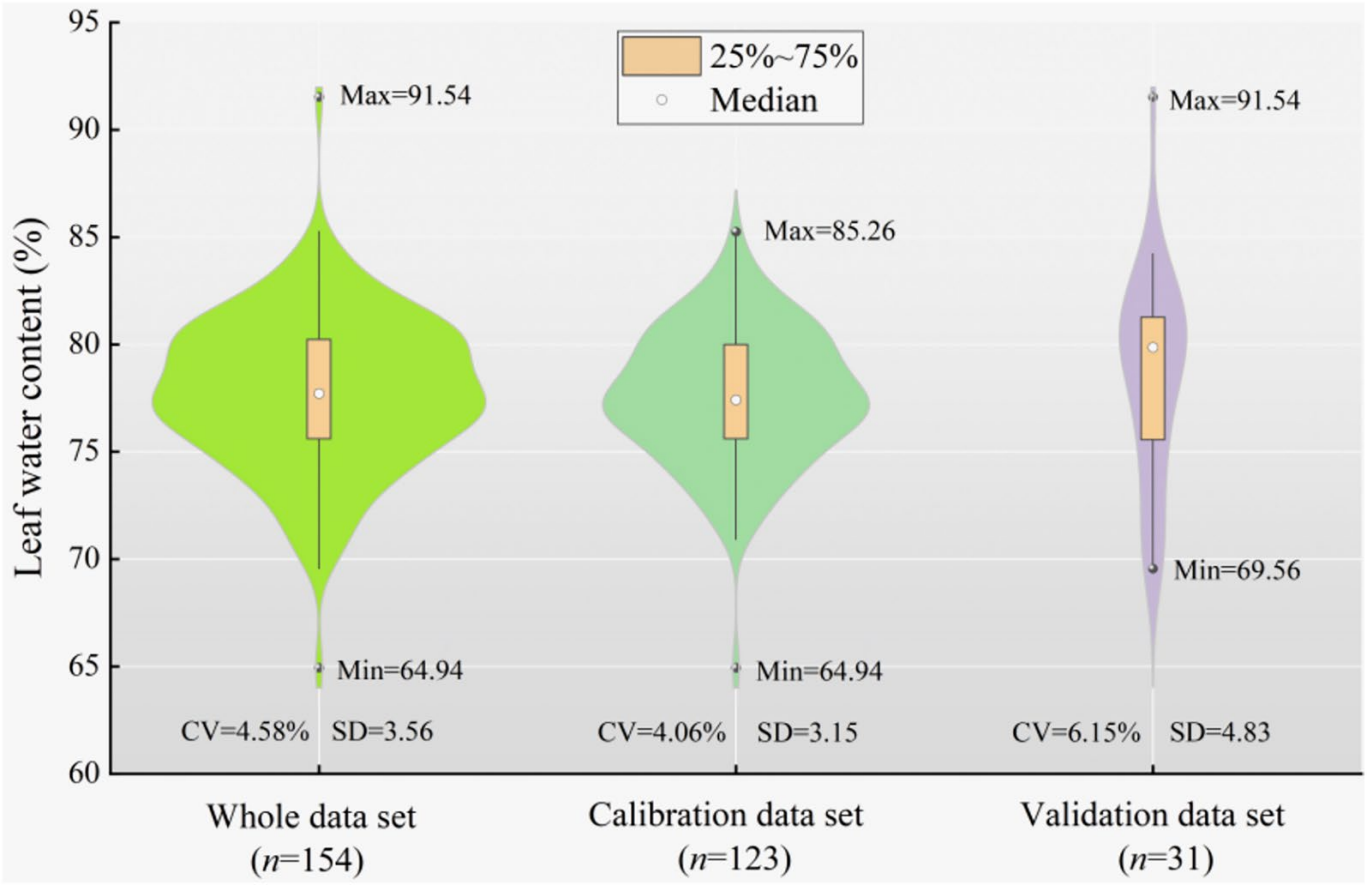
Descriptive statistics of spring wheat leaf water content. Max, Min, CV and SD represent the maximum, minimum, coefficient of variation and standard deviation, respectively

Figure 4 displays the statistical characteristics of the measured LWC for the entire dataset, including the calibration and validation subsets. The analysis reveals that the whole dataset exhibited a wide variation, with a minimum, maximum, and coefficient of variation (CV) of 64.94%, 91.54%, and 4.58%, respectively, indicating a diverse range of soil samples in the study area. The range of LWC contents observed in the validation dataset was consistent with that of the calibration dataset. The mean, standard deviation (SD), and CV values from these three datasets were relatively similar, suggesting that the calibration and validation subsets are representative of the entire population.

**Fig. 5:**
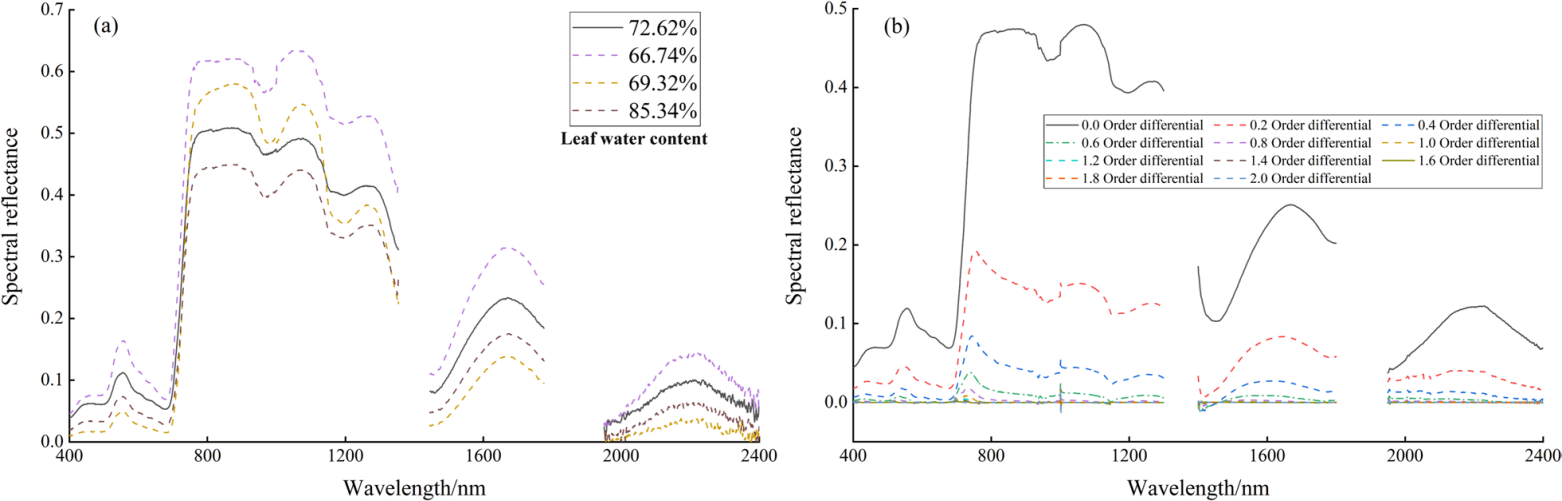
Canopy spectral curves of spring wheat. **a** Canopy spectral curves of spring wheat based on different water contents. **b** Canopy spectra of spring wheat with 0.0–2.0 order differentials

The spectral profiles of the spring wheat canopy corresponding to different moisture contents of leaves are presented in Fig. 5a. The reflectance changed due to the difference in leaf water content, and a decreasing trend was observed in spectral reflectance with an increase in leaf water content. The slopes at 930–970 nm and 1100–1200 nm increased with an increase in leaf water content. Among them, wavelengths of approximately 970 nm and 1200 nm were considered better choices for estimating leaf water content due to the absence of atmospheric disturbance.

Figure 5b shows the canopy spectral profiles of spring wheat treated with differentiation at order 0–2. Each order of differential spectral profiles had a gradual process with an increase in order, resulting in a decrease in spectral reflectance. The commonly used first- and second-order differentiation of spectral profiles differs significantly from the original one, and the information between them may be missed. On the other hand, fractional differentiation can exploit intermediate information, which can further extract and utilize hyperspectral information.

### One-dimensional correlation analysis based on FOD

**Fig. 6:**
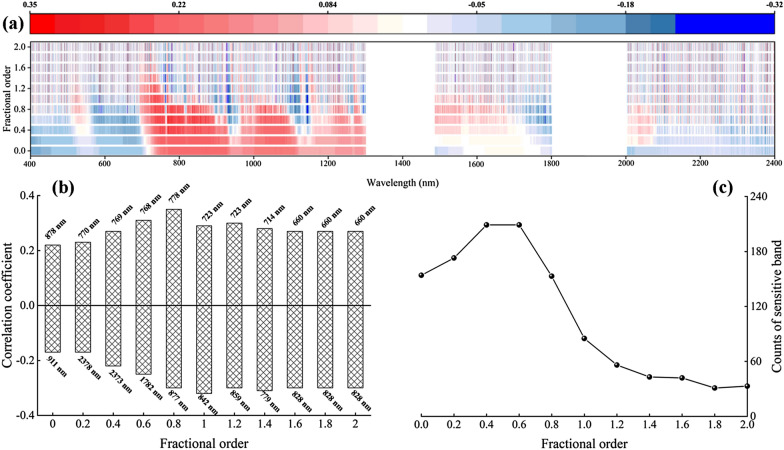
Color map of correlation coefficients (*r*) and statistical analysis. **a** Correlation coefficients between LWC and spectral reflectance based on 0.0 to 2.0 order differentials. **b** Maximum-minimum correlation coefficient for each order. **c** Number of bands with correlation coefficients tested by 0.01 level significance

Based on the above analysis, a Pearson correlation analysis was conducted on the water content of spring wheat leaves and 0–2 order differential reflectance, resulting in the distribution of correlation coefficients at each wavelength. As illustrated in Fig. 6a, the differential spectral curves of each order displayed a gradual change with increasing differential order, whereas the correlation coefficient curves exhibited increasing fluctuations and lacked strong regularity. The number of bands whose correlation coefficients passed the 0.01 level significance test displayed an initial increasing and then decreasing trend.

The 0–0.8 order differential spectral curves displayed little fluctuation and similar shapes. The number of bands with correlation coefficients gradually increased by passing the 0.01 level significance test (critical value ± 0.201), mainly concentrated in the range of 700–1100 nm, and exhibited positive and negative correlations with leaf water content (Fig. 6a). The maximum correlation coefficient appeared at 778 nm (Fig. 6b) using a 0.8-order differential, reaching 0.35. The fluctuation of 1.0–2.0 order differential spectral curves gradually increased, and the number of bands with correlation coefficients passing the 0.01 horizontal significance test gradually decreased (Fig. 6c), with the absolute values of correlation coefficients decreasing gradually. The absolute values of the correlation coefficients displayed an initial increase and then a decreasing trend, with the peak value appearing in the 0.8-order differential spectral reflectance. This finding indicates that the best order between 400 and 2400 nm is the 0.8 order.

The above results show that the fractional differential of the spring wheat canopy spectrum has some advantages in screening sensitive bands compared with the commonly used first- and second-order differentials and can more accurately find the bands that have a higher correlation with LWC.

### Two-dimensional correlation analysis based on FOD

The correlation between 12 traditional moisture indices and LWC is shown in Table 3. The analysis showed that the water vegetation index and LWC had a significant correlation, both passing the 0.01 significance level (*ρ* < 0.01), and the correlation coefficient is between − 0.22 and 0.33. The correlation coefficient between the FWBI and LWC is 0.33**, which means that the correlation between the FWBI and LWC is higher than that of the traditional water spectral index. The results show that the performance of the traditional water spectral index varies with geographic environment. Therefore, it is necessary to find a better spectral vegetation index for sensitive combinations.

**Table 3:** Correlation coefficient between moisture indices and LWC

Variable	Correlation coefficient	Variable	Correlation coefficient
WI	+ 0.22**	MSI-1	0.24**
WBI	− 0.22**	NDI	0.20**
FWBI	− 0.33**	NDVI	0.23**
SRWI-1	+ 0.18*	NDWI	0.19*
SRWI-2	+ 0.25**	NDWI-Hyp	0.21**
MSI	− 0.20**	NDMI	0.13

** and * represent the 0.01 level and 0.05 level, respectively

**Fig. 7:**
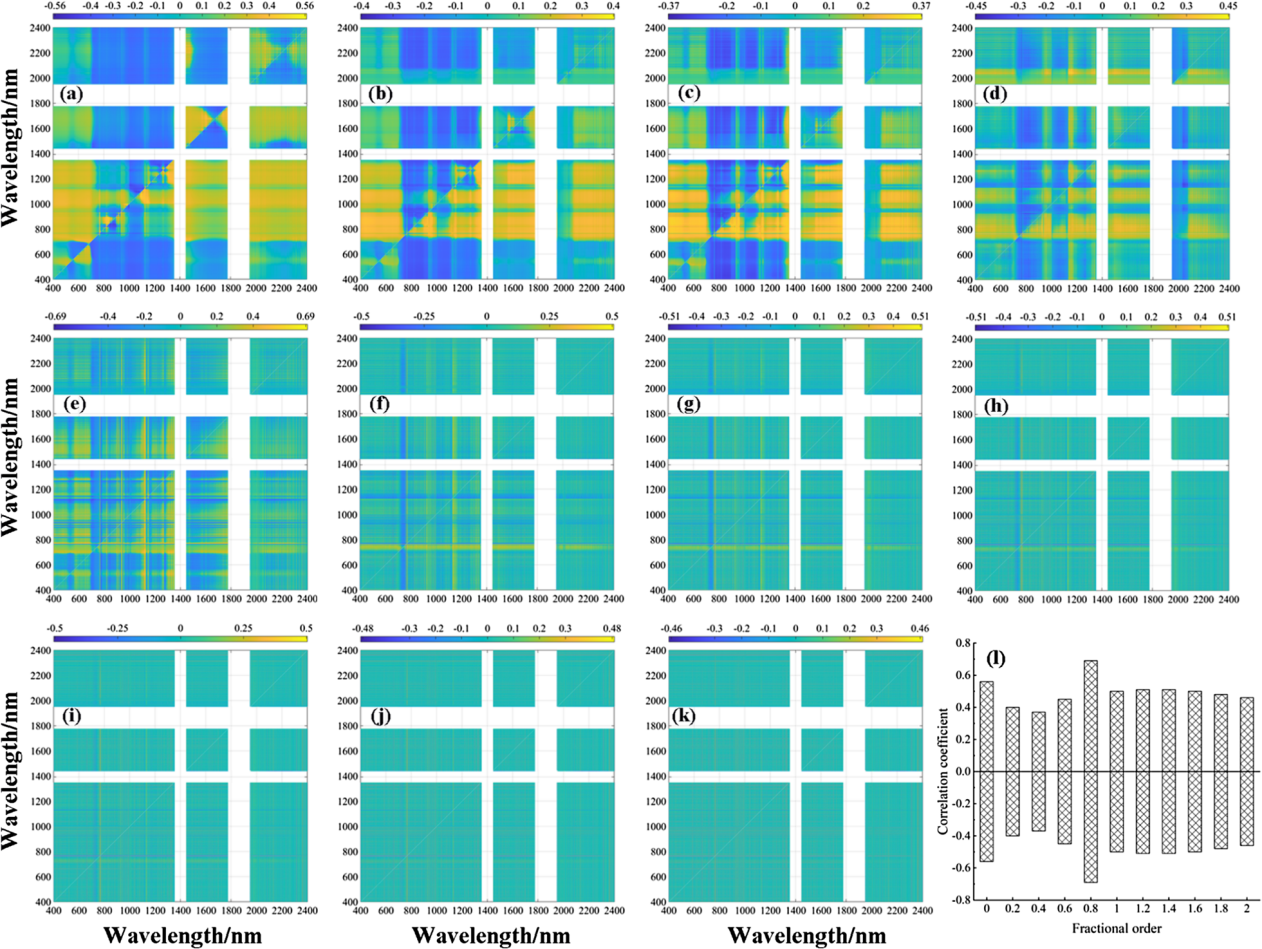
Two-dimensional correlation coefficients (*r*) between LWC and DVI in the training dataset at eleven derivative orders. **a** Raw data, **b** 0.2-order, **c** 0.4-order, **d** 0.6-order, **e** 0.8-order, **f** 1.0-order, **g** 1.2-order, **h** 1.4-order, **i** 1.6-order, **j** 1.8-order, **k** 2.0-order, and **l** maximum–minimum correlation coefficient for each order

Pearson correlation analysis was conducted between the DVI and LWC utilizing fractional differential processing, and the results are presented in the form of a heatmap in Fig. 7. An analysis of Fig. 7I. revealed that the DVI calculated at each order displayed varying degrees of sensitivity to LWC. Specifically, the correlation coefficient of the DVI to LWC calculated at orders 0–0.8 first decreased and then increased, reaching its highest correlation at order 0.8 with an absolute correlation coefficient value of 0.69. For calculations at orders 1–2, the correlation coefficient of DVI on LWC gradually decreased, and the absolute values of the correlation coefficient ranged from 0.46 to 0.69.

**Fig. 8:**
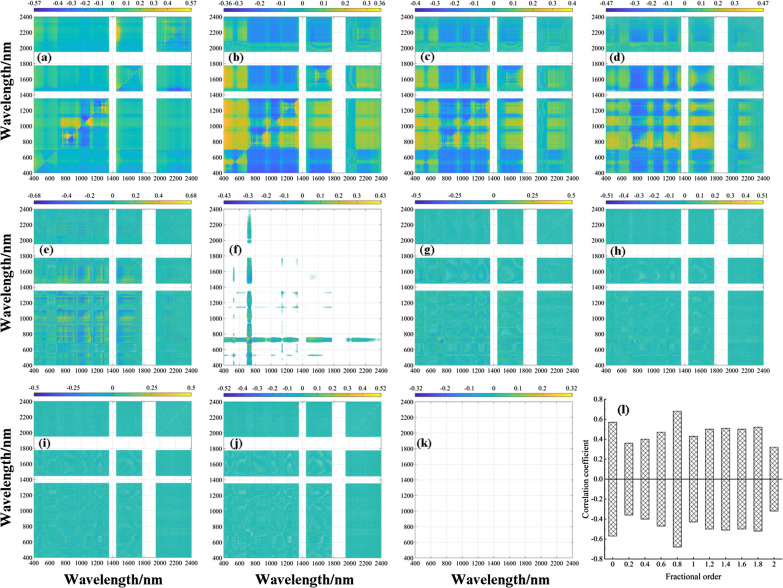
Two-dimensional correlation coefficients (*r*) between the LWC and NDVI in the training dataset at eleven derivative orders. **a** raw data, **b** 0.2-order, **c** 0.4-order, **d** 0.6-order, **e** 0.8-order, **f** 1.0-order, **g** 1.2-order, **h** 1.4-order, **i** 1.6-order, **j** 1.8-order, **k** 2.0-order, and **l** maximum–minimum correlation coefficient for each order. (Note: the reason for the white areas in Fig. 11-k is due to limited correlation data and a dispersed distribution, making it difficult to discern the presence of data in the mapping results)

**Fig. 9:**
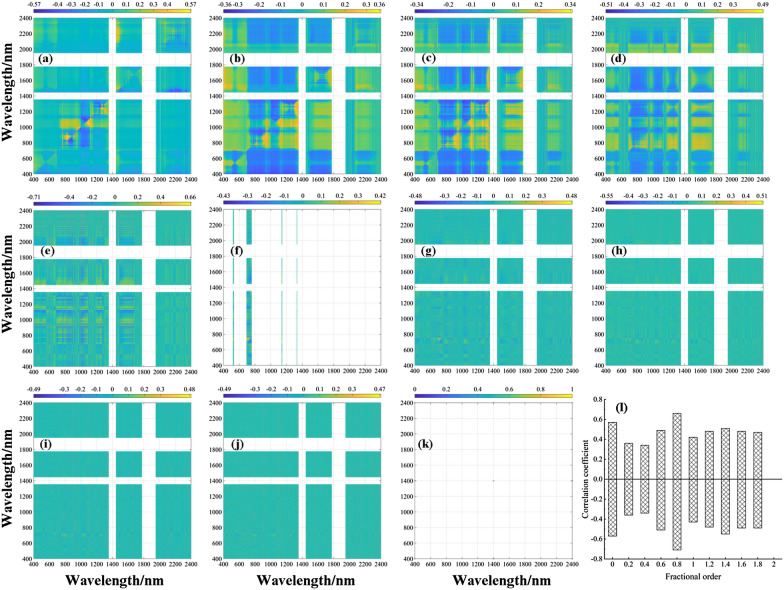
Two-dimensional correlation coefficients (*r*) between LWC and RVI in the training dataset at eleven derivative orders. **a** Raw data, **b** 0.2-order, **c** 0.4-order, **d** 0.6-order, **e** 0.8-order, **f** 1.0-order, **g** 1.2-order, **h** 1.4-order, **i** 1.6-order, **j** 1.8-order, **k** 2.0-order, and **l** maximum–minimum correlation coefficient for each order (the reason for the white areas in Fig. 12k is due to limited correlation data and a dispersed distribution, making it difficult to discern the presence of data in the mapping results)

An analysis of Figs. 8I and 9I revealed that the NDVI and RVI, respectively, calculated at each order exhibited varying degrees of sensitivity to LWC. Specifically, the NDVI and RVI calculated at orders 0–0.8 showed a decreasing trend followed by an increasing trend in the correlation coefficient of LWC and displayed the highest correlation at order 0.8, with absolute correlation coefficients of 0.68 and 0.71, respectively. For calculations at orders 1.0–2.0, the correlation coefficients of the NDVI and RVI for LWC gradually decreased, with absolute correlation coefficient values ranging from 0.32 to 0.68 and 0.34 to 0.71, respectively. The above analysis indicates that the RVI, NDVI, and DVI calculated using 0 to 0.8-order spectral reflectance presented higher correlation coefficient in Figs. 7, 8I, 9. The band combination tested for significance at 0.01 mainly focused on the range of 400–1300 nm, suggesting that this band region can provide more band combination information for changes in leaf moisture. Therefore, the next step is to utilize the 400–1300 nm band information to calculate the three-band combination vegetation index and examine its sensitivity trend to LWC.

### Three-dimensional correlation analysis based on FOD

**Fig. 10:**
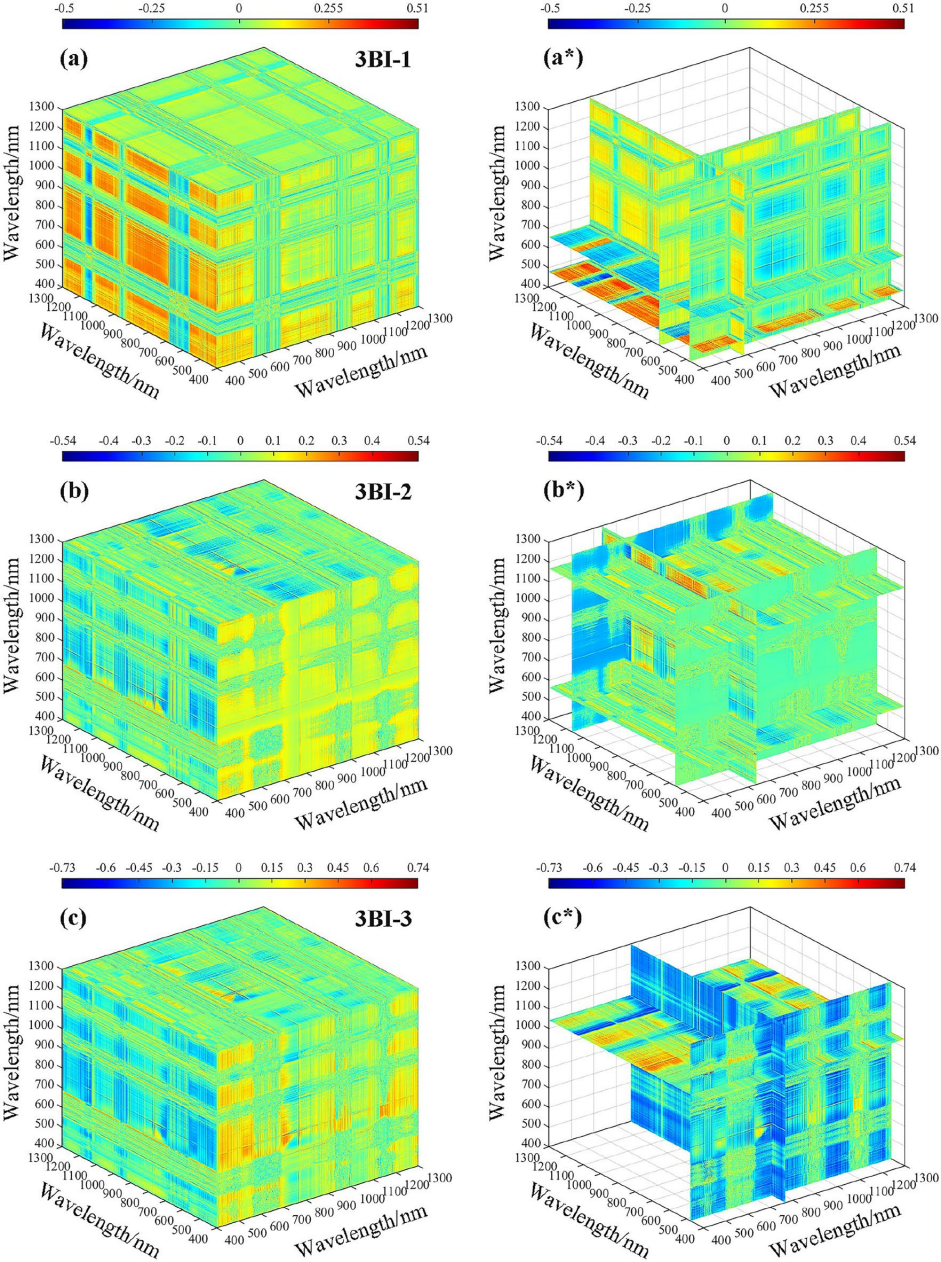

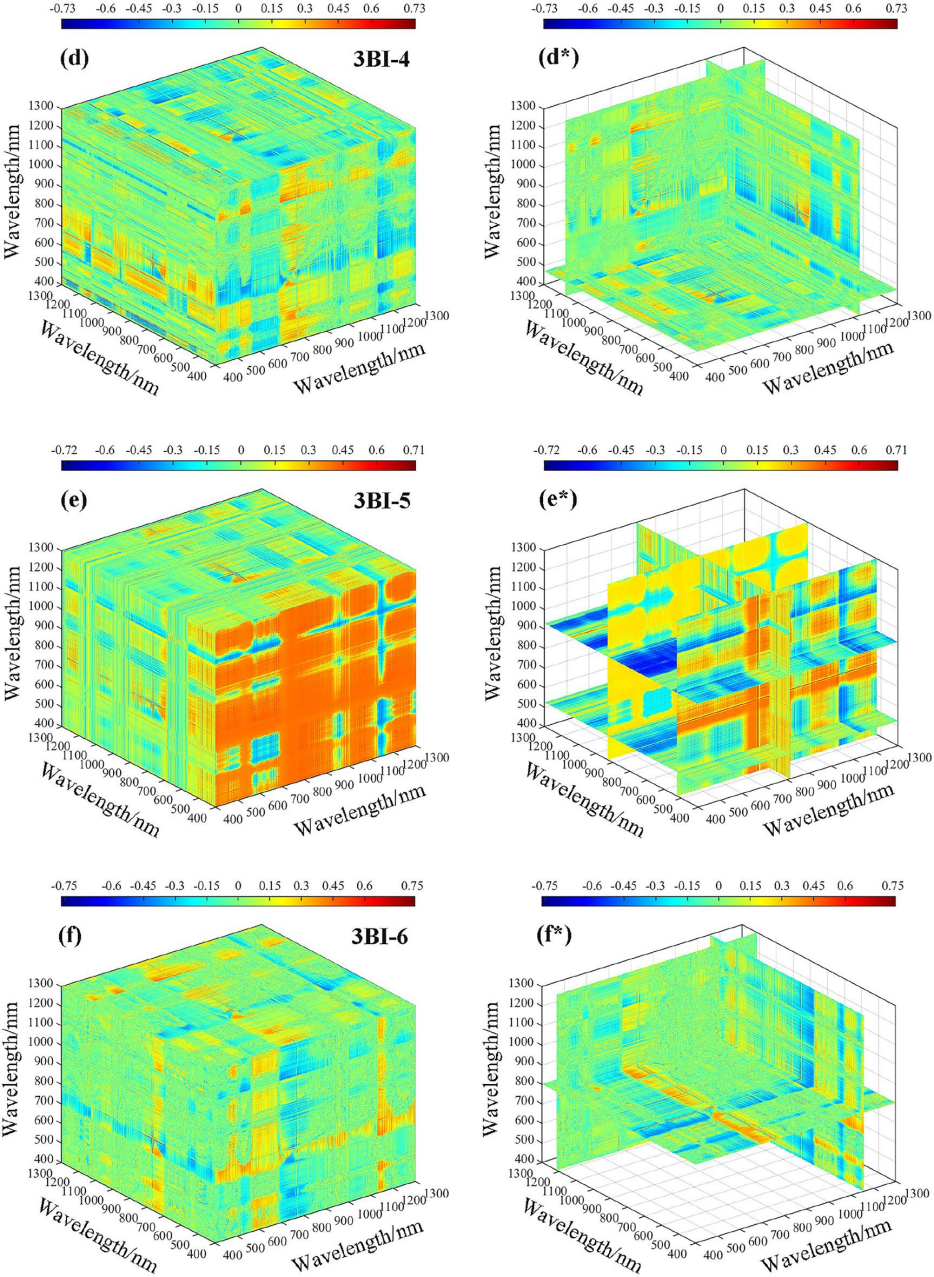

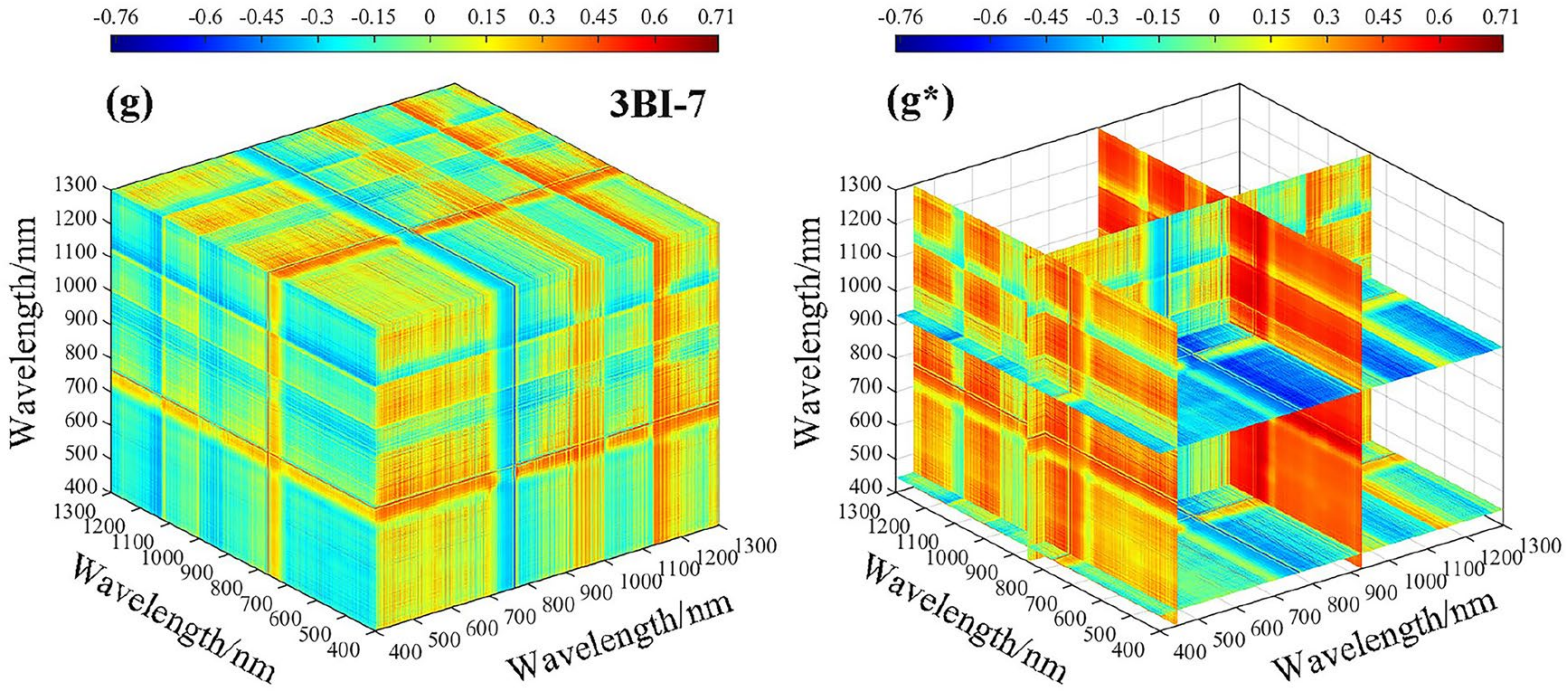
Three-dimensional correlation coefficients (*r*) between the LWC and three-band indices based on 0.8 order differentials. **a** 3BI-1, **b** 3BI-2, **c** 3BI-3, **d** 3BI-4, **e** 3BI-5, **f** 3BI-6, and **g** 3BI-7

The correlation analysis between fractional-order differential spectroscopy and the spectral index revealed that spectral reflectance based on 0.8-order pretreatment exhibited the highest correlation with LWC. The sensitive range of the two-band spectral index based on the 0.8-order differential to the LWC was 400–1300 nm. To calculate the three-band spectral index, 0.8-order spectral data within the range of 400–1300 nm were selected. Figure 10**.** shows the correlation heatmap between the three-band spectral index and LWC. Specifically, Fig. 10a–g represent the correlation results between LWC and all possible combinations of three-band vegetation indices within the range of 400–1300 nm, while Fig. 10a*–g* represent the best three-band vegetation index, which is the combination of the most sensitive bands to LWC correlation.

After conducting a sensitivity analysis on three band vegetation indices with respect to LWC, the associations between 3BI-1 and 3BI-2 with LWC were ± 0.51 and ± 0.54, respectively. However, there was no significant increase in the correlation coefficient when compared to vegetation indices in the two bands. On the other hand, 3BI-3, 3BI-4, 3BI-5, 3BI-6, and 3BI-7 showed significantly higher correlations with LWC, all of which had values above 0.70. Among them, 3BI-7 exhibited the highest correlation with LWC, with an absolute coefficient value of 0.76. Overall, the correlation coefficient showed some improvement when compared to vegetation indices in the two bands.

Combining the data from Fig. 10, the optimal three band combination information was statistically determined, and among the operations of spectral reflectance based on the 0.8-order differential treatment for three band vegetation indices, the optimal three band combination information is shown in Table 4. The reflectance corresponding to the wavelengths (929 nm, 851 nm, and 446 nm) was calculated in the form of the (*R*_929 nm_ − *R*_851 nm_) − (*R*_851 nm_ − *R*_446 nm_) combination, which could enhance the susceptibility degree to LWC, indicating that the single band information was weaker than the result of the combined band information and that the three band vegetation index was better than the two band vegetation index in terms of the combined band information.

**Table 4:** Optimum three-band combination information statistics

Type	Fractional order	Spectral index	Optimum combination band	*r*
Three-band index (3BI)	0.8-order	3BI-1	583 nm 656 nm 479 nm	0.51
		3BI-2	636 nm 1167 nm 564 nm	0.54
		3BI-3	766 nm 478 nm 1042 nm	0.74
		3BI-4	1129 nm 1175 nm 471 nm	0.73
		3BI-5	814 nm 929 nm 525 nm	-0.72
		3BI-6	1156 nm 1214 nm 802 nm	0.75
		3BI-7	929 nm 851 nm 446 nm	-0.76

“*r”* represents the correlation coefficient

### LWC prediction model based on ML

**Fig. 11:**
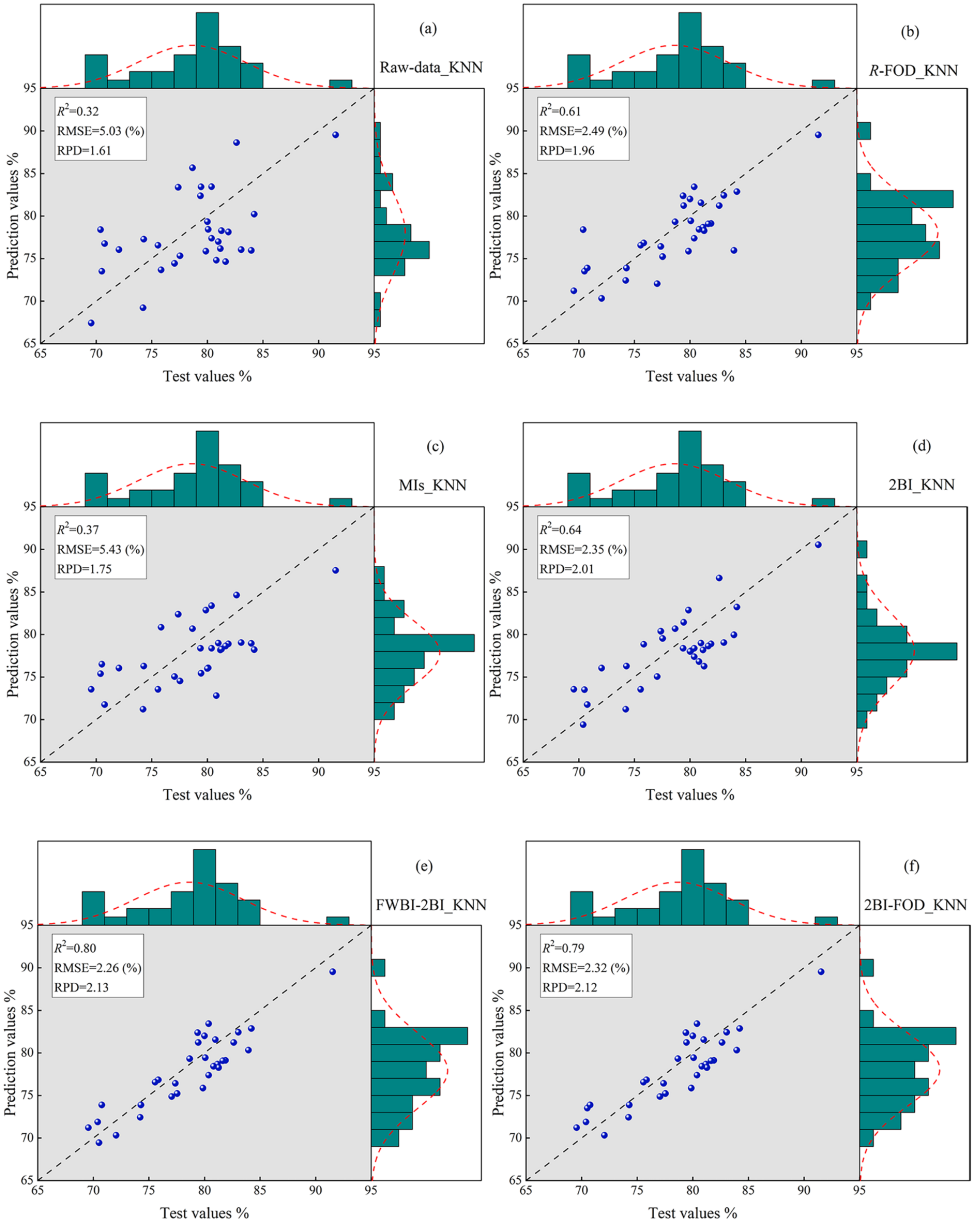

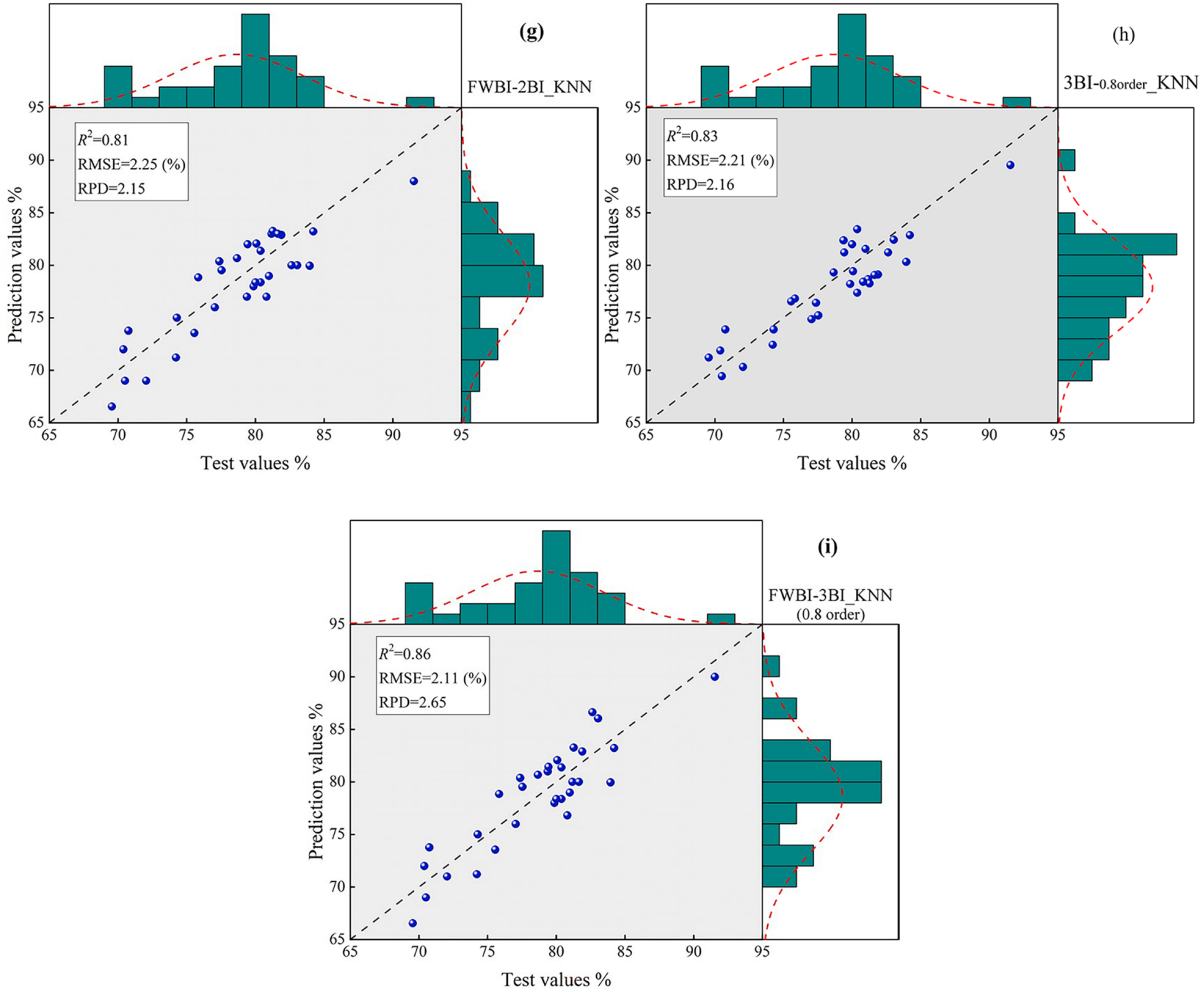
Scatter plot of measured and predicted values. **a** The KNN estimation model based on Raw-data, the wavelength (769 nm–924 nm). **b** The KNN estimation model based on *R*_-FOD_, the wavelength (711 nm–8754 nm).the FWBI and 2BI_-FOD_. **c** The KNN estimation model based on exist moisture indices. **d** The KNN estimation model using two-band optimized indices. **e** The KNN estimation model using moisture index and two-band optimized index. **f** The KNN estimation model using two-band optimized indices based on FOD. **g** The KNN estimation model using moisture index and two-band optimized indices based on FOD. **h** The KNN estimation model using three-band optimized indices based on 0.8-order. **i** The KNN estimation model based on the FWBI and three-band optimized indices based on 0.8-order

According to the correlation coefficient values and significance test (*p* < 0.01), the important wavebands were identified and extracted efficiently. K-nearest neighbor (KNN), Artificial neural network (ANN) and support vector machine (SVM) models were constructed to quantify the LWC, and an independent validation dataset was used to verify the quantitative capabilities of the models. In this study, 27 models were constructed based on machine learning, and the performances of all models are shown as scatter plots (Fig. 11).

Research shows that the model accuracy *R*^2^_*pre*_ of the sensitive single-band reflectance estimation model of LWC can reach 0.32, and the RMSE_*pre*_ is 5.03%. The model accuracy *R*^2^_*pre*_ of the spectral data after fractional order differential processing can reach 0.61, and the RMSE_*pre*_ is 2.49%. The model accuracy *R*^2^_*pre*_ of estimating LWC using the 12 existing spectral indices reached 0.37, and the RMSE_*pre*_ was 5.43%. Compared with the single-band reflectance, the model accuracy was improved. The model accuracy *R*^2^_*pre*_ of estimating with two-band optimized spectral indices (DVI, RVI and NDVI) was the highest, reaching 0.64, and the RMSE_*pre*_ was 2.35% (Table 5). The accuracy of the estimation model was significantly enhanced, indicating that optimizing the combination of spectral indices can improve the model's estimation ability.

**Table 5:** The accuracy and validation of the models based on multiband spectral data

Data-type	Variable	Method	Training dataset		Validation dataset		RPD
			*R*^2^_*sim*_	RMSE_*sim*_	*R*^2^_*pre*_	RMSE_*pre*_	
Raw-data	The reflectance of 154 corresponding to the wavelength (769–924 nm)	KNN	0.41	4.06	0.32	5.03	1.61
		SVR	0.25	5.21	0.21	5.62	1.50
		ANN	0.19	5.39	0.11	6.07	1.33
*R*_-FOD_	Reflectance of 165 at 0.8 order for wavelength 711–875 nm	KNN	0.65	2.42	0.61	2.49	1.96
		SVR	0.55	3.11	0.48	4.01	1.92
		ANN	0.46	4.18	0.37	4.56	1.84
MIs	The existing 12 moisture indices	KNN	0.46	4.12	0.37	5.43	1.75
		SVR	0.21	5.19	0.17	5.71	1.69
		ANN	0.22	4.68	0.16	6.26	1.55
2BI	DVI_(1046, 1057 nm)_ RVI_(1272, 1279 nm)_ NDVI_(1272, 1279 nm)_	KNN	0.67	2.56	0.64	2.35	2.01
		SVR	0.45	3.47	0.40	4.23	1.88
		ANN	0.39	4.21	0.28	4.86	1.76
FWBI-2BI	FWBI_(900,930,980 nm)_ RVI_(1272, 1279 nm)_	KNN	0.82	2.21	0.80	2.26	2.13
		SVR	0.78	2.28	0.74	2.32	2.09
		ANN	0.81	2.24	0.79	2.27	2.11
2BI-_FOD_	DVI_(698, 1274 nm)_ RVI_(1156, 1628 nm)_ NDVI_(1182, 1149 nm)_	KNN	0.81	2.26	0.79	2.32	2.12
		SVR	0.79	2.31	0.72	2.38	2.06
		ANN	0.81	2.29	0.78	2.34	2.08
FWBI-2BI_-FOD_	FWBI_(900,930,980 nm)_ RVI_(1156, 1628 nm)_	KNN	0.84	2.20	0.81	2.25	2.15
		SVR	0.80	2.28	0.76	2.36	2.08
		ANN	0.82	2.24	0.79	2.30	2.11
3BI_-0.8 order_	3BI-1_(583,656,479 nm)_ 3BI-2_(636,1167,564 nm)_ 3BI-3_(766,478,1042 nm)_ 3BI-4_(1129,1175,471 nm)_ 3BI-5_(814,929,525 nm)_ 3BI-6_(1156,1214,802 nm)_ 3BI-7_(929,851,446 nm)_	KNN	0.85	2.18	0.83	2.21	2.16
		SVR	0.81	2.23	0.79	2.35	2.11
		ANN	0.83	2.20	0.80	2.24	2.13
FWBI-3BI_-0.8 order_	FWBI_(900,930,980 nm)_ 3BI-7_(929,851,446 nm)_	KNN	0.89	2.05	0.86	2.11	2.65
		SVR	0.85	2.24	0.83	2.26	2.13
		ANN	0.86	2.19	0.84	2.21	2.19

“*sim*” stands for simulation, “*pre*” stands for prediction

After 0.8 spectral processing, the model accuracy *R*^2^_*pre*_ of the constructed three-band spectral index estimation reached 0.83, and the RMSE_*pre*_ was 2.21%. The model accuracies *R*^2^_*pre*_ of FWBI + 2BI-_FOD_ and FWBI + 3BI-_0.8_ reached 0.81 and 0.86, respectively, and the RMSE_*pre*_ values were 2.25% and 2.11%, respectively. The existing spectral index and the MBSI index based on fractional order differentiation significantly improved the estimation accuracy and reduced the error.

The results of the accuracy verification of the estimated LWC model constructed via sensitive bands, the existing moisture indices, two-band and three-band optimized indices. Screening was conducted on 27 estimation models constructed for 9 data types, and the results of the best estimation models corresponding to each data type were added to the part. Scatter plots of measured and predicted values were showed in Fig. 11. Among the 27 models, the models based KNN and FOD algorithm showed better prediction ability. In this study, the FWBI-3BI-0.8 order based on KNN illustrated the highest *R*^2^ accuracy, the lowest error (RMSE) and the greatest estimation ability (RPD).

## Discussion

Hyperspectral fulfilled the characteristics of multiple bands, strong continuity, and large information [64]. However, it was easy to produce overfitting problems, which can affect the predictive performance of models [65]. Therefore, in the Vis–NIR analysis of spring wheat properties, it is important to seek efficient methods to process the raw spectrum and reduce the number of redundant bands has become an important focus [65, 66].

Spectral derivative preprocessing techniques can remove baseline drift effects, reduce overlapping spectral bands, solve overlapping peaks, improve spectral resolutions and sensitivities, and eliminate interferences resulted from other background factors [31]. In previous studies, integer order derivative was commonly used for preprocessing the raw spectrum [49, 64, 65]. Wherein FOD could vary at a small interval to keep the spectral information changing slowly, further extract the effective information and allow us to detect more characteristics of certain spectral signal than integer order derivatives [18, 49]. And then some useful spectral information should not be ignored. The use of FOD improved correlation between LWC and spectrum, the best correlation coefficient for LWC was achieved using 0.8-order reflectance (Fig. 6b). These results provide the potential to establish a more LWC estimation model.

**Fig. 12:**
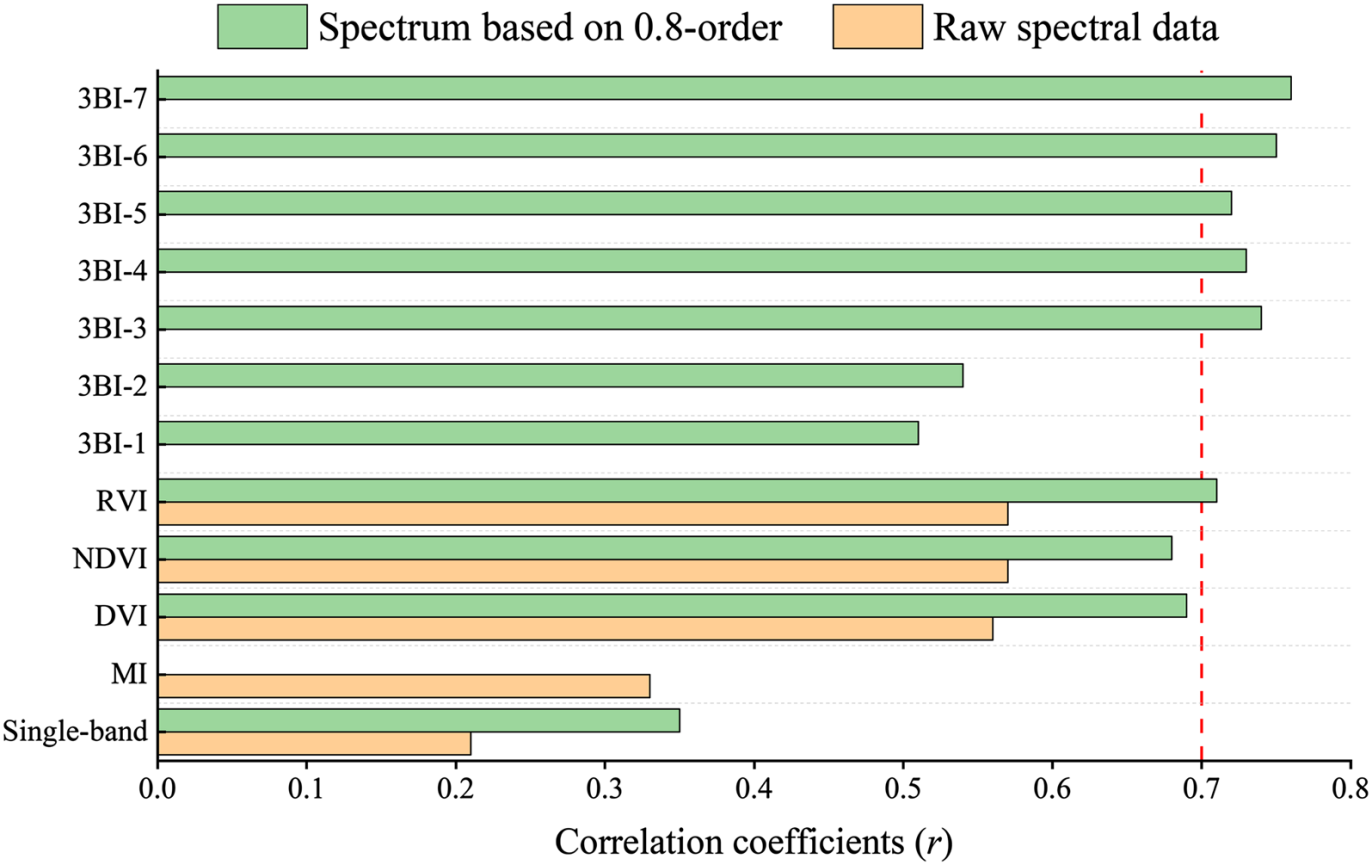
The relationship between multiband spectral data and LWC

Optimal spectral indices, which are calculated based on the sensitive wavebands related to characteristic attributes, can easily detect subtle absorption peaks and can be used to predict different spring wheat properties [63]. A number of algorithms have been proposed to optimize two-band and three-band combinations to obtain spring wheat properties of interest. In this study, we optimized the two-band and three band spectral indices based on FOD. According to the correlation analysis, the LWC data and optimized indices (two/three-band indices) have a better correlation, correlation coefficients (*r*) of RVI_(1156 nm, 1628 nm)_ was − 0.71** and 3BI-7_(929 nm, 851 nm, 446 nm)_ was − 0.76**, respectively, and the optimized indices based on FOD was significantly better than that of the exist indices (shown in Fig. 12). The results showed that there was great potential in using the band optimization algorithm and FOD to estimate LWC.

In this study, the purpose of calculating two-dimensional and three-dimensional indices is to effectively integrate relevant spectral signals and identify better band combinations. The two-band and three-band indices encompass the visible and near-infrared spectral ranges. It might be that moisture content can alter the absorption and reflection properties of leaves in the visible spectrum, while in the near-infrared spectrum, moisture content primarily affects light scattering. As leaf moisture is lost, the biological activities of the leaves are inhibited, preventing normal leaf functioning, leading to an increase in reflectance in the spectral curve of leaves in the visible and near-infrared regions as moisture loss progresses [73].

Simultaneously, we constructed 27 models for estimating the LWC in spring wheat based on multiband spectral data (single bands, moisture indices, two-band spectral indices and three-band spectral indices) and machine learning (KNN, ANN and SVR). And chosen 3 metrics to evaluate the performance of the algorithm, including *R*^2^, RMSE, and RPD. The range of *R*^2^ is [0, 1], where *R*^2^ = 1 indicates the model perfectly predicted the data, and *R*^2^ = 0 indicates the model cannot explain the variance. RMSE is a metric used to measure the predictive accuracy of a predictive model on continuous data. The value of RPD exceeds 2.0, indicating a model with better predictive ability.

The integrated index (MBSI) allows high-precision estimation of LWC, which acquired the highest coefficient of determination, the lowest root mean square error and the best predictive ability (*R*^2^ = 0.86, RMSE = 2.11% and RPD = 2.65). There is some variability in the fitting accuracy of all models, and the estimation capability of models could be ranked as follows: Model_(KNN based FWBI+3BI+0.8-order)_ > Model_(KNN based 3BI+0.8-order)_ > Model_(KNN based FWBI+2BI+FOD)_ > Model_(KNN based 2BI+FOD)_ > Model_(KNN based FWBI+2BI)_ > Model_(KNN based 2BI)_ > Model_(KNN based *R*-FOD)_ > Model_(KNN based MIs)_ > Model_(KNN based Raw data)_.

The reasons may be described as follows:

1. Spectral information is often easily affected by the leaf surface, leaf structure (lignin, cellulose, etc.) and the external environment, and the raw spectral data contains complex interference information. Simultaneously, the spectral information has a certain regional nature due to the variety and type of plants. Therefore, the estimation accuracy of single-band reflectance and the existing 12 moisture indices is different.
2. The spectral data can be refined by fractional differential processing and highlight the hidden information, which is helpful for screening the most sensitive bands, and therefore, the two-band and three-band optimized spectral indices can highlight the most influential indicators in the region, thus eliminating noise information and obtaining a more effective models.

Overall, our results show that there is great potential in using the FOD and ML to estimate LWC, and the optimal model accuracy was comparable to those reported from studies. Alireza Sharifi et al. used the Sentinel-1 SAR data and three methods (MLR, RVR and SVR) to estimate rice parameters. The results indicated that the nonparametric methods (SVR and RVR) is much better than that of the parametric regression (MLR) for rice parameter estimations [78]. Alireza Sharifi et al. used Transformed chlorophyll absorption in reflectance index (TCARI) and Modified chlorophyll absorption in reflectance index (MCARI) to determine crop nutrition status. The results indicated that the performance of TCARI and MCARI was allowed the creation of high accuracy crop nutrition maps, the use of the near infra‐red and red‐edge bands led to better results [79]. The findings of this study are similar in comparison, as the band combinations in this study also focus on the near-infrared and shortwave infrared regions, highlighting the performance ability of spectral indices. Previous research on the estimation of the moisture content of crop leaves from visible and near-infrared reflectance was shown in Table 6. After analyzing the methods of constructing models and predicting accuracy, four models, Model-_WC+FOD+ANN_, Model-_RVI437,466 nm+NDVI747,1956 nm+BPNN_, Model-_x-LW+KNN_ and the integrated Model-_MBSI+MI+FOD+KNN_, were found to have higher estimation capabilities. Except for the comparative Model-_RVI437,466 nm+NDVI747,1956 nm+BPNN_, Model-_MBSI+MI+FOD+KNN_ has higher estimation accuracy. Thus, the best performance of Model-_MBSI+MI+FOD+KNN_ can realize the regional-scale monitoring of wheat canopy water status. Xuenan Zhang et al. used a machine algorithm of gradient boosted decision tree (GBDT) based on the combination of ND_(1287,1673)_ and crop water stress index (CWSI), the optimal prediction accuracy (*R*^2^ = 0.86, RMSE = 0.01) of rice LWC was produced. In previous studies, few researchers have considered extending the spectral index method to more than two bands, particularly optimizing three-band indices and estimating crop leaf moisture by combining traditional spectral indices.

**Table 6:** Comparison of the estimation of LWC in wheat using hyperspectral data

Study area	Year	Methods	*R*^2^	References
Changping Beijing, China	2021	WI-4 + NDWSI-4 + Linear regression	0.82	[8]
Jiaozuo City, Henan, China	2023	WC + FOD + ANN	0.86	[9]
Yangzhou, China	2021	RVI_437,466 nm_ + NDVI_747,1956 nm_ + BPNN	0.88	[2]
Luoyang Henan, China	2021	x-LW + KNN	0.84	[11]
Fukang Xinjiang, China	2019	FD + GRA + PLSR	0.81	[13]
Astaneh-ye Ashrafiyeh region in the north of Iran	2019	MLR, RVR, SVR	0.92	[78]
he cities of Ray and Karaj, near the capital of Iran	2020	TCARI, MCARI	0.83	[79]
Anhui province ofChina	2024	ND_(1287,1673 nm)_ and GBDT	0.86	[80]
Fukang Xinjiang, China	–	MBSI + MI + FOD + KNN	0.86	This work

*WC* wavelet coefficients, *BPNN* BP neural network, *x-LW* x-loading weight, *WVI* water vegetation index, *GRA* grey relational analysis, *PLSR* partial least squares regression

Currently, domestic and foreign research has achieved certain results, demonstrating the feasibility of using hyperspectral technology to monitor crops in farmlands. Previous studies often used complex algorithms to construct estimation models and screen feature bands. For instant, the precision of estimating LWC in winter wheat by combining stepwise regression method and partial least squares (SRM-PLS) or PLS based on the relational degree of grey relational analysis (GRA) between water vegetation indexes (WVIs) and LWC [67, 68]. However, such algorithms have poor operability and are not conducive to obtaining feature bands intuitively and efficiently. In this study, the computational complexity of our proposed algorithm are slightly more easy; we extracted moisture-sensitive bands by preprocessing the raw spectrum using spectral information from different leaf positions and canopy layers to select the optimal spectral characterization information for wheat moisture status. We used the optimal modeling method for feature band selection to construct the optimal wheat moisture estimation models. Moreover, we achieved good results in the sensitivity band screening of the wheat leaf moisture spectral estimation model, the influence of the number of modeling bands on the accuracy of the estimation model, and the monitoring of moisture spectra in different leaf positions. This result indicates that in future research, it will be necessary to develop and utilize spectral pretreatment techniques to reduce the spectral response of LWC in order to achieve the purpose of rapid and nondestructive estimation of other spring wheat property parameters. Esmaeili et al. proposed the band selection method based on CNN embedded GA CNNeGA, the evaluation of the proposed method and the obtained results are satisfactory [81]. This method also provides strong assistance for our future screening of hyperspectral data and spectral indices.

Limitations of this study: Spring wheat exhibits different hyperspectral characteristics in different regions due to the influence of climate, leaf size, shape, growth period and other factors, therefore, many factors must be considered when selecting the leaf moisture sensitivity index of spring wheat. First, the data collected from 154 sampling points is limited, and further increasing the data volume is necessary to ensure the stability and applicability of the estimation model. Second, this study is limited to the heading stage of wheat, and it is necessary to validate the three-band indices and models in each growth stage, lacking systematic data research and modeling. Addressing these limitations will be a key focus of future research in this study.

## Conclusions

In this study, the fractional order derivative and machine learning methods were applied for estimating LWC of spring wheat, and results showed the integrated Model-_3BSMI+FOD+KNN_ achieved high accuracy. The two-band index and three-band index were extracted from spectrum based on FOD, and the linear regression analysis was conducted between these indices and LWC values. The fractional derivative pretreatment of spectral data enhances the implied information of the spectrum, and both spectral and these indices were closely correlated with the LWC values. The machine learning method for LWC estimation of spring wheat based on sensitive spectral and indices. The results showed the proposed method by combing two-band indices and three-band indices improved the estimation accuracy. The moisture content of plant leaves is an important indicator for measuring the water status of plants. Accurately estimating leaf moisture content is of great significance for studying plant physiology, ecology, agricultural production, and environmental protection. KNN, ANN and SWR were independently conducted to predict LWC values based on optimal combinations. The results showed that KNN performed better than ANN, SWR with higher *R*^2^and lower RMSE. Therefore, the results confirm that Model-_3BSMI+FOD+KNN_ is significantly effective in inverting the leaf water content of spring wheat.

## Data Availability

Data available on request. The data uderlying this article will be shared on reasonable request to the corresponding author.
